# An Overview of Systematic Reviews and Meta-Analyses on Acupuncture for Post-Acute Stroke Dysphagia

**DOI:** 10.3390/geriatrics4040068

**Published:** 2019-12-08

**Authors:** Zi-Yu Tian, Xing Liao, Ying Gao, Shi-Bing Liang, Chong-Yang Zhang, De-Hao Xu, Jian-Ping Liu, Nicola Robinson

**Affiliations:** 1 Centre for Evidence Based Chinese Medicine, Beijing University of Chinese Medicine, Beijing 100029, China; 20180941093@bucm.edu.cn (Z.-Y.T.); 20180931359@bucm.edu.cn (S.-B.L.); liujp@bucm.edu.cn (J.-P.L.); 2 Dongzhimen Hospital, Beijing University of Chinese Medicine, Beijing 100700, China; gaoying973@126.com (Y.G.); 20180931358@bucm.edu.cn (C.-Y.Z.); zyi20126185@163.com (D.-H.X.); 3 Center of Evidence Based Traditional Chinese Medicine, Institute of Basic Research In Clinical Medicine, China Academy of Chinese Medical Sciences, Beijing 100700, China; 4 School of Basic Medicine, Shanxi University of Chinese Medicine, Taiyuan 030000, China; 5 School of Health and Social Care, London South Bank University, London SE1 OAA, UK

**Keywords:** acupuncture, dysphagia, post-acute stroke, overview, systematic reviews, meta-analyses, rehabilitation

## Abstract

**Background:** Many randomized controlled trials (RCTs) and systematic reviews (SRs) on acupuncture treatment for post-acute stroke dysphagia have been published. Conflicting results from different SRs necessitated an overview to summarize and assess the quality of this evidence to determine whether acupuncture is effective for this condition. The aim was to evaluate methodological quality and summarizing the evidence for important outcomes. **Methods:** Seven databases were searched for SRs and/or meta-analysis of RCTs and quasi-RCTs on acupuncture for post-acute stroke dysphagia. Two authors independently identified SRs and meta-analyses, collected data to assess the quality of included SRs and meta analyses according to the Preferred Reporting Items for Systematic Reviews and Meta-Analyses (PRISMA) and the revised Assessment of Multiple Systematic Reviews (AMSTAR 2). **Results:** Searches yielded 382 SRs, 31 were included. The quality of 22 SRs was critically low, five SRs were low, and four Cochrane SRs were moderate when evaluated by AMSTAR2. A total of 17 SRs reported 85.2–96.3% of PRISMA items. Five SRs included explanatory RCTs, 16 SRs included pragmatic RCTs, and 10 SRs included both. Conclusion: Currently, evidence on the effectiveness of acupuncture on post-acute stroke dysphagia is of a low quality. The type of study appeared to have no direct influence on the result, but the primary outcome measures showed a relationship with the quality of SRs. High quality trials with large sample sizes should be the focus of future research.

## 1. Introduction

Stroke is considered to be one of the leading causes of adult mortality and disability [1], and the lifetime risk of stroke occurs in approximately 25% of adults over 25 years old [2]. Dysphagia is a common complication that occurs in 37% to 78% of stroke survivors who experience various problems such as eating slowly, having difficulty swallowing when drinking water, and these issues are often accompanied with a speech disorder [3]. Additionally, pneumonia, chest infection or even death may occur as a result of dysphagia [3,4]. Due to the lack of direct treatment available for post-acute stroke dysphagia (surgery or medicine), early screening for these patients is recommended to prevent the subsequent development of pneumonia, chest infection or death after stroke [5]. Screening methods cannot work alone, it depends on the accuracy and reliability of the equipment and the experience of doctors, but there is still a need to integrate care management approaches [6]. Increased risk of pneumonia following post-acute stroke dysphagia may be associated with failures in dysphagia screening after an acute stroke [7], suggesting potentially further increased costs.

Under these circumstances, patients experiencing dysphagia after a stroke may seek other approaches. As a widely used therapy in China for post-acute stroke rehabilitation, acupuncture is used in routine clinical practice for post-acute stroke dysphagia. Many clinical randomized controlled trials (RCTs) of acupuncture for post-acute stroke dysphagia have been published. Additionally, there are more than 20 systematic reviews (SRs) and meta analyses of RCTs published on acupuncture treatment for this condition. There is no doubt that SRs and meta-analyses are considered to be the gold standard to assess the effects of healthcare interventions but the current SRs on acupuncture for dysphagia after stroke show different and conflicting results. A Cochrane review published in 2016 [8] showed that acupuncture could improve swallowing function as measured by a drinking test. Another Cochrane review in 2012 [9] reported that acupuncture can reduce the prevalence of dysphagia, alleviate clinical symptoms such as difficulty swallowing and meanwhile improve the life quality of the patients. However, the latest updated Cochrane review on swallowing therapy, which included an analysis of acupuncture failed to show the improvement of swallowing ability, although heterogeneity was high [10]. Overviews of SRs and meta analyses can be performed to synthesize these studies to determine whether acupuncture is an effective treatment, thus it is necessary to conduct an overview focusing on the SRs and meta analyses of acupuncture for dysphagia after stroke.

## 2. Methods

The protocol for this study has been registered (PROSPERO registration number: CRD42019134163) and the full protocol published in the European Journal of Integrative Medicine [11].

### 2.1. Eligibility Criteria

As RCTs are considered to provide the gold standard to assess the effects of healthcare interventions, we included SRs and meta analyses of RCTs or quasi-RCTs on patients who received acupuncture treatment for post-acute stroke dysphagia based on the framework of patients/interventions/comparison-outcomes-study design (PICOS) [12]. The interventions included acupuncture/electro-acupuncture alone or combined with other treatments (including placebo, routine therapy, western medicine or rehabilitation training). Comparison treatments were defined as sham-acupuncture, herbal, routine therapy, western medicine or rehabilitation training. The primary outcomes were objective, effect-related outcomes such as: fiberoptic endoscopic examination of swallowing (FEES) or a video fluoroscopic swallowing study (VFSS) which can assess the swallowing ability. Secondary outcomes included death or the water swallow test.

### 2.2. Search Strategy for Identification of SRs and Meta Analyses

A total of seven electronic databases of published SRs and meta analyses were searched (from their inception to 27 May 2019): PubMed, EMBASE (Excerpta Medica Database), Cochrane library, China National Knowledge Infrastructure (CNKI), Wanfang Database, SinoMed Database (including China Biology Medicine disc, CBM) and China Science Technology Journal Database (VIP).

### 2.3. Study Selection

**Figure 1:**
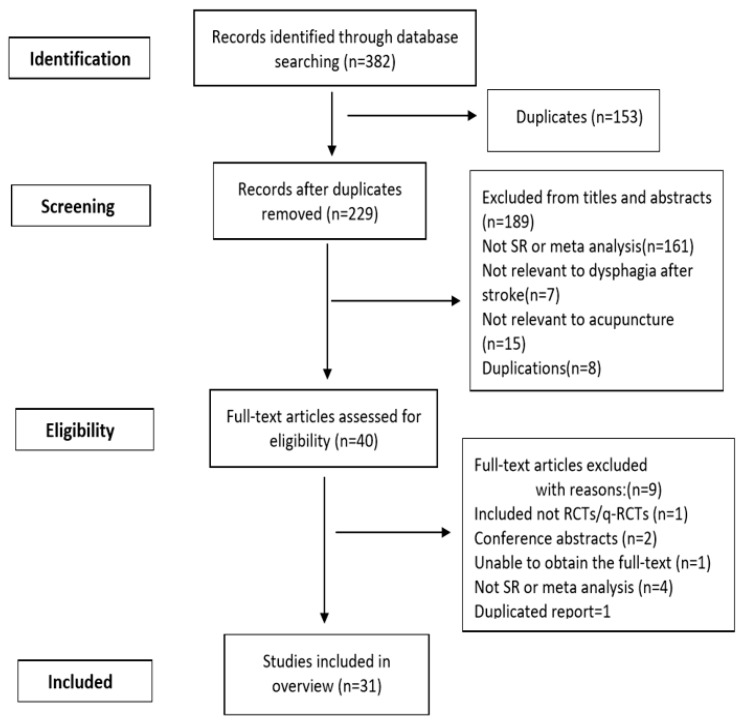
Flow diagram of literature selection.

Two reviewers identified studies according to the eligibility criteria by screening abstracts, their titles and their full text (See Figure 1 in the protocol [11]). Any disagreements were resolved through discussion and consultation with a third author.

### 2.4. Data Extraction and Synthesis

Two reviewers respectively extracted the information of included SRs and meta analyses according to the predefined Excel data extraction tables. Any disagreements were resolved through discussion and consultation with a third author. Quantitative data synthesis was not performed due to significant heterogeneity and the same primary studies being included in different SRs.

### 2.5. Quality Assessment

Two authors separately evaluated the quality of included SRs by using the Assessment of Multiple Systematic Reviews 2 (AMSTAR2) [12], which is used to assess the SRs irrespective of whether they contain RCTs or non-RCTs. Any discrepancies in the ratings of the 16 items of AMSTAR 2 were resolved by discussion and adjudication by a third author. Meanwhile, included SRs and meta analyses were also assessed by the Preferred Reporting Items for Systematic Reviews and Meta-Analyses (PRISMA) statement [13]. Any discrepancies between the two authors on the 27 items of PRISMA were resolved by discussion or judged by a third author. All included SRs used the Grading of Recommendations Assessment, Development and Evaluation (GRADE), the Cochrane risk of bias tool or Jadad score as a quality assessment tool, and for most included SRs the Efficacy rate (ER) was used as the outcome but the concept of ER was different among these studies, and most studies did not clarify the definition of ER, we did not assess the evidence quality of this outcome by GRADE.

## 3. Results

### 3.1. Selection of the Systematic Reviews and Meta Analyses

The search identified 382 articles, including 12 from PubMed, 60 from VIP, 31 from EMBASE, 15 from the Cochrane Library, 31 from CNKI, 111 from Sino-Med and 122 from the Wanfang Database. After 153 duplicate records were removed, the titles and abstracts of the remaining 229 records were reviewed. Of these, 189 records were removed after screening titles and abstracts as they were irrelevant, seven SRs were not relevant to post-acute stroke dysphagia, 15 SRs were not relevant to acupuncture and a further eight SRs were duplications. A total of 40 potentially relevant articles were downloaded for full-text screening. Subsequently nine studies were excluded, and the reasons for exclusion were as follows: one SR included observational studies, two articles were conference abstracts, one SR we were unable to get the full text, four studies were not SRs, and one article was a duplicate. Finally, 31 studies were included in our study [8,9,10,14,15,16,17,18,19,20,21,22,23,24,25,26,27,28,29,30,31,32,33,34,35,36,37,38,39,40,41]. The study flow chart is shown in Figure 1.

### 3.2. Characteristics of Systematic Reviews

A total of 31 studies were published between 2006 and 2019 [8,9,10,14,15,16,17,18,19,20,21,22,23,24,25,26,27,28,29,30,31,32,33,34,35,36,37,38,39,40,41], nine SRs [8,9,10,36,37,38,39,40,41] were published in English-language journals, and the remaining 22 [14,15,16,17,18,19,20,21,22,23,24,25,26,27,28,29,30,31,32,33,34,35] were in the Chinese-language. Four of the 31 SRs were Cochrane reviews [8,9,10,41], 21 were journal articles [14,15,16,17,18,19,20,21,22,23,24,25,26,27,29,30,36,37,38,39,40], one was a conference paper [28] and five were theses (three Masters and two PhDs degrees) [31,32,33,34,35]. The first authors of 29 SRs [8,14,15,16,17,18,19,20,21,22,23,24,25,26,27,28,29,30,31,32,33,34,35,36,37,38,39,40,41] were from China, including one [39] from Hong Kong, and the first author of the other two SRs were from UK [10] and Sri Lanka [9] but with the same corresponding author from UK. Only four [8,9,10,41] SRs published on Cochrane listed the evidence-based medicine background of co-authors, the remaining 27 [14,15,16,17,18,19,20,21,22,23,24,25,26,27,28,29,30,31,32,33,34,35,36,37,38,39,40] were unclear, and the number of co-authors ranged from one to nine, we were unable to get this information from the five [31,32,33,34,35] theses. As for the included study type, six [16,17,20,21,30,34] SRs included RCTs or quasi-RCTs, and 25 [8,9,10,14,15,18,19,22,23,24,25,26,27,28,29,31,32,33,35,36,37,38,39,40,41] SRs only included RCTs. The range of primary studies included in each SR varied from one to 72, and number of participants ranged from 431 to 6134. Three SRs just reported the number of included primary studies, but did not report the number of participants [8,27,31]. It was not possible to obtain available information on the ages of dysphagia patients in each SR. Regarding the types of intervention, five SRs [9,10,20,25,40] included acupuncture treatment alone, 13SRs [8,19,21,22,23,24,26,27,28,33,36,38,41] included acupuncture combined with other therapies (including rehabilitation or swallowing training, medicine, baseline treatment), 13 SRs [14,15,16,17,18,29,30,31,32,34,35,37,39] included both acupuncture treatment and acupuncture treatment combined with other therapies (including rehabilitation or swallowing training, medicine, baseline treatment). As for the control group, 24 SRs [14,15,17,18,20,21,23,24,25,26,27,28,29,30,32,33,34,35,36,37,38,39,40,41] compared acupuncture with rehabilitation training, medicine or conventional treatment, there were no limitations on treatment of the control group in two studies [16,31], two Cochrane SRs used no treatment, acupuncture on different points or sham acupuncture as their control group [9,10], while both rehabilitation training or medicine alone or plus routine or sham acupuncture were used as control treatments in the other three studies [8,19,22]. Primary outcomes were heterogeneous being defined differently in all included SRs. Efficacy rate (ER) and water swallow test (WST) were used frequently. Video fluoroscopic swallowing study (VFSS), standardized swallowing assessment (SSA), fiber optic endoscopic examination of swallowing (FEES), swallowing function assessment (SFA), death or dependency were also used as primary outcomes. All studies used a quality assessment tool, only two Cochrane SRs [8,10] used Grades of Recommendation, Assessment, Development and Evaluation (GRADE) to evaluate the quality of the evidence. A total of 20 SRs [9,14,16,17,19,20,24,25,26,28,29,30,31,32,33,34,35,36,37,41] used Cochrane risk of bias tool, six SRs [15,18,21,22,27,38] used the Jadad score, one SR [23] used both Cochrane risk of bias tool and Jadad score, one SR [39] used Cochrane risk of bias tool and Physiotherapy Evidence Database (PEDro) scale and only one SR [40] used Consolidated Standards of Reporting Trials (CONSORT) and the Revised Standards for Reporting Interventions in Clinical Trials of Acupuncture (STRICTA) checklist to evaluate the included RCTs.

A total of 10 SRs [10,23,30,31,32,33,35,36,37,40] mentioned adverse events (fewer than 3% occurred in each SR), while only five SRs [10,31,36,37,40] reported adverse events that were associated with acupuncture, and four SRs [23,30,32,35] reported pain, ecchymosis and hematoma during the process or at the site of needling, while the other SR [33] reported the pain occurred after electro acupuncture. These data may suggest that acupuncture rarely caused serious side effects, and that acupuncture could be considered as safe for the treatment of post-acute stroke dysphagia. All included SRs concluded that there was very low to low quality evidence on the effectiveness of acupuncture treatment on post-acute stroke dysphagia, there is still a need for high quality trials with large sample sizes. Table 1 lists the characteristics of these included SRs.

### 3.3. Quality of the Systematic Reviews

#### 3.3.1. Methodological Quality Assessed by AMSTAR2

**Figure 2:**
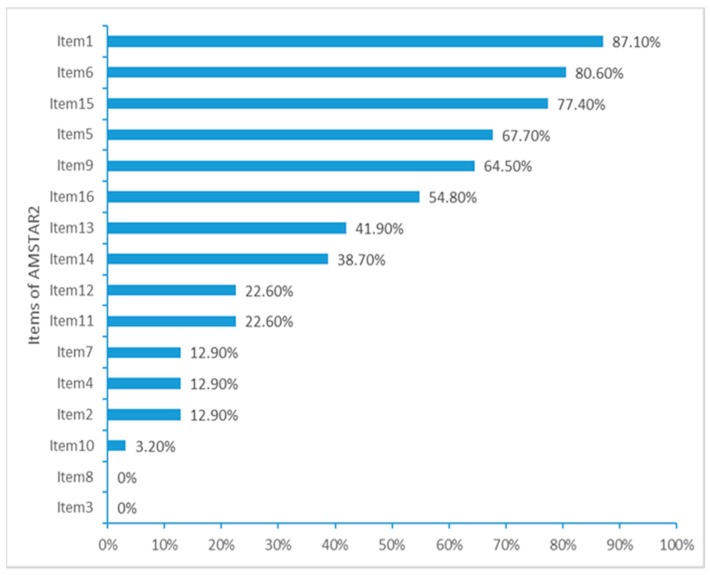
Total percentage yes for each item of AMSTAR 2 (‘yes’: items are answered completely and met the requirements of the sub-items, ‘no’: items are absent or evaluation is inappropriate, or ‘partial yes’: only some of the items answered).

**Figure 3:**
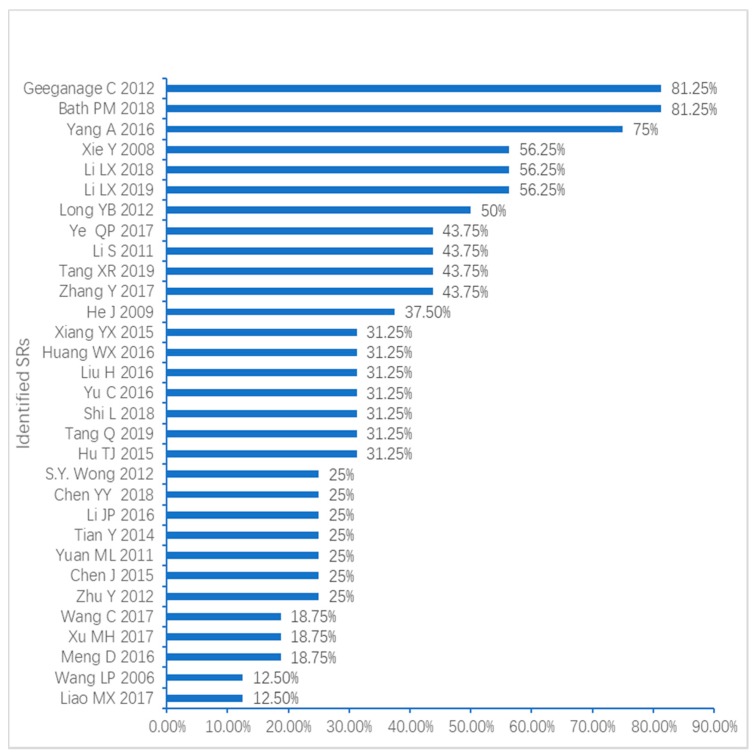
The percentage yes for each identified SRs assessed by AMSTAR 2 (‘yes’: items are answered completely and met the requirements of the sub-items, ‘no’: items are absent or evaluation is inappropriate, or ‘partial yes’: only some of the items answered).

The overall quality of 22 SRs [14,15,16,17,18,19,20,21,22,23,24,25,26,27,28,29,30,31,32,33,34,35] (71%) were rated critically low, five SRs [36,37,38,39,40] (16.1%) were low, while four Cochrane SRs [8,9,10,41] (12.9%) were moderate. Only seven SRs [8,9,10,36,38,40,41] (22.6%) reported over half of the 16 items on AMSTAR 2, while the remaining 23 SRs [14,15,16,17,18,19,20,21,22,23,24,25,26,27,28,29,30,31,32,33,34,35,37] (77.4%) reported just less than eight items on AMSTAR 2. The components of PICO (patient, intervention, control group and outcome) for item 1 in research questions and inclusion criteria were well reported in 27 SRs [8,9,10,16,17,19,20,21,22,23,25,26,27,28,29,30,31,32,33,34,35,36,37,38,39,40,41] (87.1%), but no SR mentioned the time frame for follow-up. Only four Cochrane SRs [8,9,10,41] (12.9%) reported the predefined protocol (critical item 2), but there were no SRs that provided reasons for including only RCTs (item 3). The four Cochrane SRs [8,9,10,41] (12.9%) provided a systematic searching strategy, while the remaining 27 SRs [14,15,16,17,18,19,20,21,22,23,24,25,26,27,28,29,30,31,32,33,34,35,36,37,38,39,40] (87.1%) just achieved the partial searching on databases (item 4). As for item 5 and item 6, most of SRs identified eligible studies and extracted data by two reviewers (respectively 67.7% [8,9,10,14,17,19,22,23,24,26,28,29,32,33,35,36,37,38,39,40,41], and 80.6% [8,9,10,14,16,17,18,19,20,22,23,24,26,28,30,31,32,33,34,35,36,37,38,39,40,41]). Only the four Cochrane SRs [8,9,10,41] (12.9%) provided a list of all excluded studies, while other SRs [14,15,16,17,18,19,20,21,22,23,24,25,26,27,28,29,30,31,32,33,34,35,36,37,38,39,40] (87.1%) provided none or just part of potentially relevant full-text excluded studies (item 7). There were no SRs reporting the timeframe for follow-up (item 8). Although all the studies used Jadad score or Cochrane risk of bias tool to evaluate the quality of evidence, just 20 SRs [8,9,10,14,15,17,18,20,23,24,25,26,27,29,31,32,33,34,35,41] (64.5%) assessed the overall risk of bias (item 9). Just one Cochrane SR [10] (3.2%) reported the source of funding (item 10). Two SRs [39,41] did not conduct meta analysis, only 7 SRs [8,9,10,36,37,38,40] (22.6%) combined RCTs results using appropriate techniques and investigated the causes of heterogeneity on item 11. For the assessment of the impact of risk of bias of individual studies on the results of the data synthesis, item 12, the results were the same as that of item 11. But 13 SRs [8,9,10,14,18,20,23,26,36,37,38,40,41] (41.9%) took the risk of bias into consideration when discussing the results of the SRs (item 13). A total of twelve SRs [8,9,10,14,18,21,25,36,38,39,40,41] (38.7%) showed no significant heterogeneity in the results or investigated the source of heterogeneity in the results and discussed their effect on the results of the study (item 14). A total of 24 SRs [8,9,10,14,15,17,18,19,20,21,22,23,24,26,27,28,29,31,32,33,34,35,36,40] (77.4%) showed the results of possible publication bias (small study effects) with funnel plot in detail. Further, a total of 17 SRs [9,14,15,16,19,20,21,22,23,24,26,28,29,36,37,38,40] (54.8%) reported the conflict of interest. The AMSTAR 2 checklist is listed in Table S2 and assessment results of the AMSTAR 2 are provided in Table S3, Figure 2 and Figure 3. (Table S2: AMSTAR 2 checklist; Table S3: methodological quality assessment by AMSTAR 2)

#### 3.3.2. Reporting Quality Assessed by PRISMA

No SR reported all 27 items of PRISMA, three SRs [25,38,39] (9.7%) reported 40–51.9% items of PRISMA, 11 SRs [15,17,18,20,21,24,26,27,29,30,37] (35.5%) reported 66.7–85% items and 17 SRs [8,9,10,14,16,19,22,23,28,31,32,33,34,35,36,40,41] (54.8%) reported 85.2–96.3% items. The majority of SRs [8,9,10,14,15,16,17,18,19,20,21,22,23,25,26,27,28,29,30,31,34,35,36,37,38,39,40,41] (90.3%) identified the report as a SR, meta-analysis, or both in the title. All SRs provided structured summaries, but just four Cochrane SRs [8,9,10,41] (12.9%) registered their protocol. For items 3, 6, 7, 24, 26 all 100% reported on the 31 included SRs. Item 3 describes the rationale of the studies in introduction section, item 6 focuses on eligibility criteria of the studies including the characteristics of studies and reporting (e.g., language limitation, grey literature or publication status), providing a rationale at the same time. Item 7 describes the process of searching the databases with date, while item 24 and item 26 contains a summary of current evidence in discussion section and interpretation of the study results for clinical practice or research in the future in the concluding section. As for the PICO, which should be contained in the objectives, 25 SRs [8,9,10,14,15,16,17,18,19,20,21,22,23,24,25,26,27,28,29,30,31,32,33,34,35] (80.6%) gave an explicit statement in the introduction or eligibility criteria section. Four Cochrane SRs [8,9,10,41] provided the protocol, and two SRs [37,40] (19.4% in total) indicated predefined protocol, but just one provided an address that could be accessed. In the methods section, 18 SRs [8,9,10,14,16,19,20,22,27,28,30,31,32,33,35,36,40,41] (58%) provided searching strategy of at least one database. A total of 23 SRs [8,9,10,14,18,19,22,23,24,27,28,29,31,32,33,34,35,36,37,38,39,40,41] (74.2%) reported the process for selecting studies and 27 SRs [8,9,10,14,16,17,18,19,20,22,23,24,25,26,27,28,31,32,33,34,35,36,37,38,39,40,41] (87.1%) stated the process for data extraction. A total of 19 SRs [8,9,10,16,18,19,22,24,26,27,31,32,33,34,36,38,39,40,41] (61.3%) stated data items and 23 SRs [8,9,10,14,16,17,20,22,23,24,28,30,31,32,33,34,35,36,37,38,39,40,41] (74.2%) reported the assessment of risk bias of individual studies, 28 SRs [8,9,10,14,15,16,17,18,19,21,22,23,24,25,26,27,28,29,30,31,32,33,34,35,36,38,40,41] (90.3%) summarized the outcome measures and the same percentage of SRs reported [8,9,10,14,15,16,17,18,19,20,21,22,23,24,25,27,28,29,30,31,32,33,34,35,36,38,40,41] the method of synthesizing the results. A total of 22 SRs [8,9,10,14,17,18,19,20,21,22,23,26,28,29,30,31,32,35,36,38,40,41] (71%) assessed other bias like publication biases, and 23 SRs [8,9,10,14,15,17,18,19,22,23,26,28,30,31,32,33,34,35,36,37,38,40,41] (74.2%) conducted sensitivity or subgroup analyses, but only the four Cochrane SRs [8,9,10,41] provided predefined additional analyses. In the results section, 29 SRs [8,9,10,14,16,18,19,20,21,22,23,24,25,26,27,28,29,31,32,33,34,35,36,37,38,39,40,41] (93.5%) gave information on the study selection, only one SR [25] (3%) did not list the characteristics of included RCTs, 21 SRs [8,9,10,14,16,17,22,23,28,30,31,32,33,34,35,36,37,38,39,40,41] (67.7%) presented information of data on risk of bias of included studies. One SR [39] (3%) did not list all outcomes of each study. Apart from two SRs [39,40] that did not conduct meta analysis, 29 SRs [8,9,10,14,15,16,17,18,19,20,21,22,23,24,25,26,27,28,29,30,31,32,33,34,35,36,37,38,41] (93.57%) provided results of each meta-analysis with confidence intervals. A total of 22 SRs [8,9,10,14,17,18,19,20,21,22,23,26,28,29,30,31,32,35,36,38,40,41] (71%) reported results of publication risk of bias across studies, and 21 SRs [8,9,10,16,17,18,19,20,23,26,27,30,31,32,33,34,35,36,37,38,41] (67.7%) conducted sensitivity or subgroup analyses.

In conclusion, 20 SRs [8,9,10,14,15,16,21,22,28,29,31,32,33,34,35,36,37,38,40,41] (64.5%) mentioned the limitations of their study, and the summary of the evidence and conclusions were 100% reported. Finally, 14 SRs [14,15,16,19,20,21,22,23,24,26,28,29,36,37] (45.2%) reported sources of funding for the SRs. The PRISMA checklist and assessment results of the PRISMA are presented in Tables S4 and S5. (Table S4: PRISMA checklist; and Table S5: reporting quality assessment by PRISMA).

### 3.4. Comparison Types of Acupuncture

In all 31 SRs, five SRs [9,10,20,25,40] included explanatory RCTs, 13 SRs [8,19,21,22,23,24,26,27,28,33,36,38,41] included pragmatic RCTs, and 13 SRs [14,15,16,17,18,29,30,31,32,34,35,37,39] included both explanatory RCTs and pragmatic RCTs. The types of comparisons in the 18 SRs [8,9,10,19,20,21,22,23,24,25,26,27,28,33,36,38,40,41] are summarized as follows.

#### 3.4.1. Explanatory RCTs

##### Acupuncture Versus No Treatment/Sham Acupuncture/Routine Acupuncture

Two Cochrane SRs used no treatment, acupuncture on different points or sham acupuncture as their control group [9,10], data from four studies in one SR [9] showed a reduction in dysphagia by end of the trial (t = 4; n = 256; odd ratio (OR) = 0.24; 95% confidence interval (CI) [0.13, 0.46]; *p* < 0.0001; I^2^ = 0%), there was no difference in swallow scores between treatment and control groups. However, significant heterogeneity was noted (t = 3; n = 256; mean difference (MD) = −0.41; 95% CI [−1.53, 0.72]; I^2^ = 91%; *p* < 0.0001) for swallow scores in acupuncture studies. Subgroup analysis in another Cochrane SR [10] showed that acupuncture (OR = 0.31; 95% CI [0.20, 0.49]; 676 participants; eight studies; I² = 0%; *p* < 0.00001) reduced dysphagia at end of trial. However, these findings may be due to chance, given that testing for subgroup differences did not yield significant results. Acupuncture did not reduce swallowing ability SMD (Standardized Mean Difference) = −0.55; 95% CI [−1.20, 0.11]; 496 participants; six studies; I² = 91%; *p* = 0.10).

##### Acupuncture Versus Rehabilitation Training/Western Medicine/Routine Therapy

Three SRs [20,25,40] compared acupuncture alone with rehabilitation training/conventional therapy, one SR [20] showed that ER and overall recovery rate in acupuncture group were higher than rehabilitation training or routine therapy (relative risk (RR) = 1.38; 95% CI [−1. 28, 1.49]; *p* < 0.01; RR = 2. 56; 95% CI [ 2.15, 3.04]; *p* < 0.01; 37 studies; 3697 participants); ER in acupuncture group was higher than other therapies in one SR [25] (OR (Total) = 3.97; 95% CI [2.73, 5.67]; *p* = 0.25; I^2^ = 9%; 907 participants; 11 studies); acupuncture treatment provided a higher ER compared with rehabilitation training/routine medicine ([RR = 1.33; 95% CI [1.25, 1.43]) [40].

#### 3.4.2. Pragmatic RCTs

##### Acupuncture Plus Baseline Treatment/Medicine/Rehabilitation Training Versus Baseline Treatment/Medicine/Rehabilitation Training

One Cochrane SR [8] showed that acupuncture plus baseline treatment for people with convalescent phase stroke, can improve dependency (nine trials; 616 participants; MD = 9.19; 95% CI [4.34, 14.05]; GRADE very low), global neurological deficiency (seven trials; 543 participants; OR = 3.89; 95% CI [1.78, 8.49]; GRADE low), and specific neurological impairments including motor function measured (four trials; 245 participants; MD = 6.16; 95% CI [4.20, 8.11]; GRADE low), cognitive function (five trials; 278 participants; MD = 2.54; 95% CI [0.03, 5.05]; GRADE very low) and swallowing function (two trials; 200 participants; MD = −1.11, 95% CI [−2.08, −0.14]; GRADE very low), relieved depression (six trials; 552 participants; MD = −2.58; 95% CI [−3.28, −1.87]; GRADE very low), and pain (two trials; 118 participants; MD = −2.88; 95% CI [−3.68, −2.09]; GRADE low). Another Cochrane SR [41] just included one trial of 66 participants and demonstrated that the acupuncture group showed no statistically significant differences when compared with baseline group. The relative risk of recovery was 1.61 with a 95% CI of 0.73 to 3.58. One SR [19] compared different stage of stroke, for stroke in the convalescent phase, the ER for acupuncture plus baseline treatment group was higher than the baseline control group [RR = 1.45; 95% CI [1.16, 1.80]; *p* = 0.001; eight studies; 766 participants), but there was no difference at the acute stage.

Nine SRs [21,22,23,24,28,33,36,38] compared acupuncture plus rehabilitation training with rehabilitation alone. Four SRs [21,27,33,36] showed that acupuncture can improve ER (*p* < 0.01. OR = 3.84; 95% CI [2.91, 5.05]; *p* < 0.00001; RR = 1.26; 95% CI [1.19, 1.34]; *p* < 0.001; 14 studies. RR=1.26; 95% CI [1.11, 1.43]; P < 0.00001; 12 studies; 1172 participants), and the other four SRs [22,23,24,28,38] defined ER as the improvement of VFSS, SSA and WST scores demonstrating that acupuncture can improve ER (OR = 3.66; 95% CI [2.66, 5.05]; *p* < 0.00001; 17 studies. RR = 1.22; 95% CI [1.16, 1.28]; *p* < 0.01; 16 studies; 1780 participants. OR = 3.80; *p* < 0.00001; 95%CI [2.58, 5.60]; 12 studies; 1012 participants. OR = 2.9; 95% CI [2.16, 3.91]; *p* = 1.00; 15 studies; 1229 participants. OR = 5.17, 95% CI [4.18, 6.38]; *p* < 0.00001; 32 studies; 6134 participants). When compared to drugs, one SR [26] showed that the ER and the overall recovery rate for acupuncture combined with drugs was higher than drugs alone (RR = 1.73, 95% CI [1.37, 2.20]; RR = 3.30, 95%CI [2.07, 5.25], seven studies, 1187 participants).

## 4. Discussion

Acupuncture is used as routine clinical therapy in China for post-acute stroke dysphagia. There is some evidence that it can improve the cerebral blood flow and serum levels of brain-derived neurotrophic factor (BDNF) and nerve growth factor (NGF) in dysphagia patients [42], but still there is a lack of widely agreed evidence of a biologically-plausible basis for its effect. This overview summarizes the current evidence on the effectiveness of acupuncture for post-acute stroke dysphagia. Of the 31SRs and meta-analyses identified, most were of critically low quality assessed by AMSTAR2, and almost half of them reported 85.2–96.3% items on PRISMA. Due to the very low to low quality of evidence, and insufficient reporting data provided by these SRs and meta-analyses, there is still no definitive conclusion on the effectiveness of acupuncture for post-acute stroke dysphagia.

Most SRs and meta-analyses included in this overview ignored the need to register the protocol, and no SRs and meta-analyses provided an explanation for including only RCTs, future studies should pay attention to these two items in AMSTAR 2. The included 31 SRs and meta analyses in this overview were published between 2006 and 2019, nine SRs [9,17,20,21,26,30,38,39,41] were published before 2012 and seven SRs [10,22,23,24,34,36,40] were published between 2018 and 2019. However PRISMA was published in 2009 [13] and the AMSTAR 2 was updated in 2017 [12]. Many journals require authors to self-evaluate according to the PRISMA statement when submitting a systematic review and meta-analysis. This may be one of the reasons that most of the SRs were critically low as assessed by AMSTAR 2. In addition, many SRs included trials that were conducted prior to the development and use of these two quality assessment tools. As 15 SRs [8,14,15,16,18,19,25,27,28,29,31,32,33,35,37] were published between 2013 and 2017, the increasing number of SRs focusing on acupuncture for post-acute stroke dysphagia not only indicates the interest and concern regarding effectiveness of acupuncture in this area, but it also means that SRs are widely used to assess the therapeutic effect based on the original studies. However, the quality of current SRs are low. As we know, AMSTAR is a quality assessment tool used just for SRs of RCTs, while AMSTAR 2 is an update of AMSTAR, which can be used to appraise SRs of intervention trials including both RCTs and NRCTs [12]. We can see from the 16 items of AMSTAR 2 and 27 items of PRISMA, that some items are mutually complementary. PRISMA emphasizes the structure of the SR, while AMSTAR 2 is concerned more with the details of methodology used for included original studies in the SR, especially the risk of bias (including additional bias). Item 27 in the PRISMA checklist stresses the importance of funding for the SR and availability of other support (e.g., supply of data), while AMSTAR 2 emphasizes the funding source of original studies, both of quality measures take potential conflict of interest into account, but only AMSTAR 2 specifically lists the item of conflict of interest. Therefore, the combined use of the two tools can provide an overall assessment of the quality of SRs that focus on healthcare interventions. Future SRs should conduct and report the SRs according to these two tools.

The overall quality of the four Cochrane SRs [8,9,10,41] were assessed as moderate by AMSTAR 2, and these SRs reported more than 90% items of PRISMA. But the results of these four SRs were still inconsistent. One of the Cochrane SRs [41] only included one trial of 66 participants, and compared a routine treatment combined with acupuncture with routine treatment alone, but the statistical significance regarding the primary outcome of feeding was not reported, the relative risk (RR) of recovery was 1.61 with a 95% confidence interval (CI) [0.73, 3.58]. One of the SRs [8] which included three RCTs showed that acupuncture was superior to no acupuncture in the terms of improving swallowing function as measured by the drinking test (mean difference (MD) = −1.11, 95% CI [−2.08, −0.14]; participants = 200; studies = 2; I² = 96%), and the difference of another included study was also significant (odd ratio (OR) = 95.29, 95% CI [10.93, 830.86]). However, the opposite result was reported in 2012 [9], comparing to sham acupuncture or no acupuncture, there was no difference in swallowing scores between treatment and acupuncture groups. But the heterogeneity was significant (t = 3; n = 256; MD = −0.41; 95% CI [−1.53, 0.72]; I2 = 91%; *p* < 0.0001), and the updated Cochrane SR [10] supported the result that acupuncture did not improve swallowing ability (SMD −0.55, 95% CI −1.20 to 0.11; 496 participants; six studies; I² = 91%; *p* = 0.10), still with significant heterogeneity, but acupuncture can reduce the number of participants with dysphagia at end of trial (OR = 0.31, 95% CI [0.20, 0.49]; 676 participants; eight studies; I² = 0%; *p* < 0.00001).Therefore, the quality of included RCTs was also another reason that influenced the quality of SRs.

Apart from the methodological problems of the included RCTs, there was diversity in the selection of primary outcomes. Effective rate (ER) and water swallow test were the most frequently used measures to assess the effectiveness of acupuncture on post-acute stroke dysphagia, but the concept of ER was different among these studies, and most studies did not clarify the definition of ER. Just six SRs [22,23,24,28,38,40] reported the definition of ER: effective rate= (‘recovery’ + ‘markedly improved’ + ‘improved’)/total number of patients, and ‘recovery’ meant totally cure, ‘markedly improved’ represented nearly complete resolution of dysphagia, while ‘improved’ represented partial resolution of dysphagia. One SR [40] used the change in water swallow score to assess the resolution of dysphagia, but the authors of this SR did not report how they assessed the resolution of dysphagia. This may have overestimated the efficacy of the intervention to some extent, and future research should provide clear definitions of related outcomes. Besides, just three SRs [8,19,41] mentioned the different stages of stroke, almost each SR included both cerebral hemorrhage and infarction without subgroup analysis and only one SR [40] used CONSORT and STRICTA checklist to evaluate the included RCTs, these studies provide insufficient information on what and how acupuncture was delivered. These are important factors that may influence the efficacy of acupuncture but data was unavailable from the original RCTs.

Due to some of the same primary studies being included in different SRs, and updates of SRs and meta-analyses, data synthesis was not appropriate. Although, the method used for this overview was performed according to the criteria for conducting overviews of SRs and meta analyses given in the Cochrane Handbook of Systematic Reviews of Interventions [43], it still has some limitations, as there are no clear standards for conducting an overview of systematic reviews and meta-analyses. Although a comprehensive literature search was conducted, relevant SRs may have been missed. Stroke was defined as a Mesh word, and acupuncture, dysphagia were used as key words in the title and abstract. This overview may have missed some stroke relevant studies that did not list the acupuncture as treatment or dysphagia as a symptom in the title or abstract.

## 5. Conclusions

Currently there is very low to low quality evidence on the effectiveness of acupuncture for post-acute stroke dysphagia. RCTs with high quality and large sample sizes are needed as well as SRs and meta-analyses with high quality. Although the evidence was insufficient to provide definitive conclusions on the effectiveness of acupuncture for post-acute stroke dysphagia, there are preliminary indications that it may improve symptoms associated with dysphagia. Future SRs should consider not only the reporting quality but also the methodological quality when conducting a SR, some details like the clear definition of primary outcomes, the subtype and the different stage of disease should also be considered.

## Figures and Tables

**Table 1:** Characteristics of systematic reviews.

SRs	Country (First Author)	No. of Primary Studies (Patients)	Age	Adverse Effects	Study Types	Intervention Measures		Primary Outcome(s)	Evidence Quality Evaluation Tool	Main Conclusions
						Treatment Group	Control Group			
1 Zhang Y 2017	China	12/824	Not mentioned	No	RCT	1*, 2*	3*	ER/WST	Cochrane risk of bias tool	Electro acupuncture was an effective treatment for post stroke dysphagia but still need more high quality RCTs to support this conclusion.
2 Meng D 2016	China	48/4785	Not Mentioned	No	RCT	1, 2	3	ER/WST/SSA	Jadad score	Both acupuncture treatment and acupuncture combined with swallowing function rehabilitation training are more effective in treating post stroke dysphagia (compared to other therapies).
3 Liao MX 2017	China	42/3268	Not Mentioned	No	RCT/q-RCT	1, 2	no limitation	ER/WST	Cochrane risk of bias tool	Jin’s three-needle alone or combined with other therapies can effectively improve the efficacy of pseudo-bulbar paralysis after stroke, but more RCTs with high-quality and large-sample size are needed.
4 Zhu Y 2012	China	7/701	Not Mentioned	No	RCT/q-RCT	1, 2	3	ER/SFA	Cochrane risk of bias tool + Jadad score	Acupuncture can effectively improve the efficacy of pseudo-bulbar paralysis after stroke, but more RCTs with high-quality and large-sample size are needed.
5 Hu TJ 2015	China	17/1158	Not Mentioned	No	RCT	1, 2	3	ER	Jadad score	Acupuncture with points on neck was an effective treatment for post stroke dysphagia but more high quality RCTs to support this conclusion is still needed.
6 Chen J 2015	China	8/766	49–78yrs	No	RCT	2	3 or 4*	ER	Cochrane risk of bias tool	Acupuncture combined with conventional therapy (swallowing function training, medical treatment) benefits swallowing function recovery on patients with dysphagia after stroke. The evaluation of the timing, treatment, and concurrent treatment of dysphagia after stroke still requires well designed RCTs with large-scale and high-quality.
7 He J 2009	China	37/3697	Not mentioned	No	RCT/q-RCT	1	3	ER	Cochrane risk of bias tool	The therapeutic effect of acupoint stimulating therapy on post stroke dysphagia is better than that the control group, but more well designed randomized, are needed to support this conclusion.
8 Yuan ML 2011	China	13/962	Not mentioned	No	RCT/q-RCT	2	3	ER	Jadad score	Acupuncture combined with rehabilitation training is beneficial to the recovery of swallowing function for patients with post stroke dysphagia. However, more well-designed RCTs are needed to support this conclusion.
9 Tang Q 2019	China	22/1987	46–76 yrs	No	RCT	2	3 or 4	ER/VFSS/SFA/WST	Jadad score	Acupuncture combined with rehabilitation training is effective for post stroke dysphagia and the combined effect is better than rehabilitation training alone. However, due to the small size and low quality of included RCTs, well designed RCTs with large-scale and high-quality are still required.
10 Tang XR 2019	China	16/1780	Not mentioned	pain, ecchymosis and hematoma	RCT	2	3	ER/WST/ SSA	Cochrane risk of bias tool + Jadad score	Acupuncture combined with rehabilitation therapy was an effective treatment for post stroke dysphagia but still need more high quality RCTs to support this conclusion.
11 Shi L 2018	China	12/1015	38–78yrs	No	RCT	2	3	ER/WST	Cochrane risk of bias tool	Acupuncture combined with rehabilitation training increased the efficacy and reduced the degree of dysphagia in post stroke patients but still more high quality RCTs needed to support this conclusion.
12 Xu MH 2017	China	11/907	Not mentioned	No	RCT	1	3	ER/ SSA	Cochrane risk of bias tool	Acupuncture therapy had better effects on post stroke dysphagia.
13 Li S 2011	China	7/1187	Not mentioned	No	RCT	2	3	ER	Cochrane risk of bias tool	Acupuncture therapy for pseudobulbar palsy is effective, but more high-quality RCTs are required to support this conclusion.
14 Wang C 2017	China	32/NR	Not mentioned	No	RCT	2	3	ER	Jadad score	Acupuncture treatment for post stroke dysphagia shows better clinical efficacy. Multi-center and large-sample RCTs are still needed to support this conclusion.
15 Tian Y 2014	China	15/1229	Not mentioned	No	RCT	2	3	ER/WST/ VFSS	Cochrane risk of bias tool	Acupuncture combined with swallowing training has obvious effect for post stroke dysphagia. The swallowing function of patients improved more obviously than that of the control group at the same time.
16 Yu C 2016	China	9/577	Not mentioned	No	RCT	1, 2	3	ER/WST	Cochrane risk of bias tool	Acupuncture was efficacious in treating post stroke dysphagia, but still high-quality and large-sample-size RCTs are required to support this conclusion.
17 Wang LP 2006	China	7/506	Not mentioned	Subcutaneous hemorrhage at local point	RCT/q-RCT	1, 2	3	ER, VFSS, death	Cochrane risk of bias tool	A reliable conclusion cannot be drawn from the present data because of the low methodological quality, especially because of the lack of data on long-term outcomes. A tendency that acupuncture can improve dysphagia after stroke in short--term with no adverse effect id demonstrated. Therefore, it is necessary to conduct more multi-central RCTs with high quality in future.
18 Li JP 2016	China	47/NR	Not mentioned	Mention-ed	RCT	1, 2	no limitation	ER/WST	Cochrane risk of bias tool	Jin’s three-needle was more effective than other therapies for post stroke dysphagia.
19 Liu H 2016	China	14/1155	Not mentioned	Subcutaneous hemorrhage at local point	RCT	1, 2	3	ER/WST/VFSS	Cochrane risk of bias tool	Conventional treatment plus acupuncture was more effective for some outcomes than conventional treatment alone, but RCTs with higher quality in the future may produce new evidence.
20 Huang WX 2016	China	12/1172	Not mentioned	Pain occurred after electro-acupuncture	RCT	2	3	ER/WST/ VFSS	Cochrane risk of bias tool	Acupuncture and moxibustion can improve the swallowing function of patients with post stroke dysphagia (better than rehabilitation training alone).
21 Chen YY 2018	China	4/425	Not mentioned	No	RCT/q-RCT	1, 2	3	WST	Cochrane risk of bias tool	Compared with the conventional rehabilitation treatment group, the acupuncture treatment group had better improvements for post stroke dysphagia according to the WST, but still requires high-quality and large-sample-size RCTs to support this conclusion.
22 Xiang YX 2015	China	17/1440	40–85yrs	Subcutaneous hemorrhage at local point	RCT	1, 2	3	ER/WST/ SSA/VFSS	Cochrane risk of bias tool	Acupuncture combined with drugs is better than simple drugs assessed by VFSS scores. Acupuncture combined with drugs and rehabilitation training is better than drugs combined with rehabilitation assessed by SSA and VFSS scores. Acupuncture has a positive effect on improving the WST, SSA and VFSS score. However, it has not been proven that acupuncture combined with drugs and rehabilitation training can reduce the incidence of aspiration pneumonia. Acupuncture combined with rehabilitation is better than rehabilitation training alone but acupuncture and rehabilitation training have the same effect on the treatment of patients with post stroke dysphagia. However, the long-term effect of acupuncture on post stroke dysphagia is better than rehabilitation training.
23 Li LX 2019	China	17/1479	27–78yrs	Mentioned	RCT	2	3	ER, SFA, IA, QOL	Cochrane risk of bias tool	Acupuncture combined with swallowing training can improve the ER, SFA and IAs of daily life in patients with post stroke dysphagia compared with swallowing training alone. However, further RCTs with large sample sizes and high quality are required to support this conclusion.
24 Ye QP 2017	China	71/6010	42–82yrs	Mention-ed	RCT	1, 2	3	WST, SSA, ER	Cochrane risk of bias tool	Acupuncture was better than conventional therapies in terms of efficacy rate of post stroke dysphagia. However, further RCTs with large sample sizes and high quality are required to support this conclusion.
25 Long YB 2012	China	72/6134	Not mentioned	No	RCT	2	3	ER	Jadad score	Acupuncture may be beneficial in rehabilitation of patients with post stroke dysphagia. Further high-quality RCTs are still needed.
26 S.Y. Wong 2012	China (Hong Kong)	9/783	40–88yrs	No	RCT	1, 2	3	SSA/VFSS/FEES	Cochrane risk of bias tool, PEDro scale	Definitive conclusions on acupuncture with conventional rehabilitation therapy for post stroke dysphagia cannot be made due to the low-quality evidence, but this combination approach appears to be promising. We recommend that acupuncture may still be used as combination use by qualified practitioners as it is relatively safe without much negative effect
27 Li LX 2018	China	29/2190	Not mentioned	Mention-ed	RCT	1	3	WST, KSA, FDS, VFSS, CSA	CONSORT, STRICTA	Acupuncture is an effective and safe alternative therapy for treatment to post-stroke dysphagia, although the beneficial effect from acupuncture is possibly overvalued due to the low methodology quality of the included RCTs. More high-quality and large-scale research studies are needed.
28 Xie Y 2008	China	1/66	No Limitation	No	RCT	2	3	Resolution of dysphagia (defined as recovery of normal feeding, which includes solid food and water, but does not include pureed food)	Cochrane risk of bias tool	There is not enough evidence to make any conclusion about the therapeutic effect of acupuncture for dysphagia after acute stroke. High quality and large scale randomized controlled trials are needed
29 Yang A 2016	China	4/NR	24–95	No	RCT	2	3 + 4	Death or dependency at the end of follow-up	GRADE	From the available evidence, acupuncture may have beneficial effects on improving dependency, global neurological deficiency, and some specific neurological impairments for people with stroke in the convalescent stage, with no obvious serious adverse events. However, most included trials were of inadequate quality and size. There is, therefore, inadequate evidence to draw any conclusions about its routine use. Rigorously designed, randomized, multi-center, large sample trials of acupuncture for stroke are needed to further assess its effects.
30 Bath PM 2018	UK	11/998	mean 67.8 yrs	Mentioned	RCT	1	4	Death or dependency/disability	GRADE	Moderate and low-quality evidence suggests that swallowing therapy did not have a significant effect on the outcomes of death or dependency/disability, case fatality at the end of the trial, or penetration aspiration score. However, swallowing therapy may have reduced length of hospital stay, dysphagia, and chest infections, and may have improved swallowing ability. However, these results are based on evidence of variable quality, involving a variety of interventions (including acupuncture). Further high-quality trials are needed to test whether specific interventions are effective.
31 Geeganage C 2012	Sri Lanka	4/256	Average age of patients across the studies was 71 years	No	RCT	1	4	Death or dependency, or death or disability	Cochrane risk of bias tool	Acupuncture and behavioral therapy may reduce dysphagia, although the effective components for each remain unclear. Further research is needed to discover which components of swallowing therapy, including acupuncture, are beneficial.

Notes: RCTs: randomized controlled trials. q-RCTs: quasi-RCTs. SRs: systematic reviews. PRISMA: Preferred Reporting Items for Systematic Reviews and Meta-Analyses. AMSTAR: Assessment of Multiple Systematic Reviews. PICOS: patients/interventions/comparison-outcomes-study design. PICO: patient, intervention, control group and outcome. Intervention measures: treatment group: 1* = acupuncture/electroacupuncture alone. 2* = acupuncture/electroacupuncture combined with other therapies. Control group: 3* = rehabilitation/swallowing training, medicine or baseline treatment. 4* = acupuncture on different points, sham acupuncture or no treatment. VFSS: video fluoroscopic swallowing study. GRADE: Grading of Recommendations Assessment, Development and Evaluation. ER: efficacy rate. WST: water swallow test. SSA: standardized swallowing assessment. SFA: swallowing function assessment. CONSORT: Consolidated Standards of Reporting Trials. STRICTA: Revised STandards for Reporting Interventions in Clinical Trials of Acupuncture. FEES: fiberoptic endoscopic examination of swallowing. PEDro: Physiotherapy Evidence Database. IA: individual activity. QOL: quality of life. KSA: Kubota Toshio’s swallowing ability assessment. FDS: Fujishima Ichiro’ s dysphagia scale. CSA: clinical symptoms assessment.

## Supplementary Materials

The following are available online https://www.mdpi.com/2308-3417/4/4/68/s1. Table S2: AMSTAR 2 checklist; Table S3: methodological quality assessment by AMSTAR 2; Table S4: PRISMA checklist; Table S5: reporting quality assessment by PRISMA; Figure S4: search strategy in PubMed.
