# Supplementary material for: An Overview of Systematic Reviews and Meta-Analyses on Acupuncture for Post-Acute Stroke Dysphagia
Source: Geriatrics (Basel). 2019 Dec 8;4(4):68. doi: 10.3390/geriatrics4040068 (PMC6960703; doi:10.3390/geriatrics4040068)
Supplement: Supplementary file 1 [file geriatrics-04-00068-s001.pdf]

Table 2: AMSTAR 2 Checklist

1. Did the research questions and inclusion criteria for the review include the components of PICO?		
For Yes:<br>☐ Population<br>☐Intervention<br>☐ Comparator group<br>☐ Outcome	Optional（recommended）<br>☐ Timeframe for follow-up	☐ Yes<br>☐ No
2. Did the report of the review contain an explicit statement that the review methods were established prior to the conduct of the review and did the report justify any significant deviations from the protocol?		
For Partial Yes: The authors state that they had a written protocol or guide that included ALL the following:<br>☐ review question(s)<br>☐ a search strategy<br>☐ inclusion/exclusion criteria<br>☐ a risk of bias (RoB) assessment	For Yes: As for partial yes, plus the protocol should be registered and should also have specified:<br>☐ a meta-analysis/synthesis plan, if appropriate, and<br>☐ a plan for investigating causes of heterogeneity<br>☐ justification for any deviations from the protocol	☐ Yes<br>☐ Partial Yes<br>☐ No
3. Did the review authors explain their selection of the study designs for inclusion in the review?		
For Yes, the review should satisfy ONE of the following:<br>☐ Explanation for including only RCTs<br>☐ OR Explanation for including only NRSI<br>☐ OR Explanation for including both RCTs and NRSI		☐ Yes<br>☐ No
4. Did the review authors use a comprehensive literature search strategy?		
For Partial Yes (all the following):<br>☐ searched at least 2 databases (relevant to research question)<br>☐ provided key word and/or search strategy	For Yes, should also have (all the following):<br>☐ searched the reference lists/bibliographies of included studies<br>☐ searched trial/study registries	☐ Yes<br>☐ Partial Yes<br>☐ No

☐ justified publication restrictions (e.g. language)	☐ included/consulted content experts in the field<br>☐ where relevant, searched for grey literature<br>☐ conducted search within 24 months of completion of the review	
5. Did the review authors perform study selection in duplicate?		
For Yes, either ONE of the following:<br>☐ at least two reviewers independently agreed on selection of eligible studies and achieved consensus on which studies to include<br>☐ OR two reviewers selected a sample of eligible studies and achieved good agreement (at least 80 percent), with the remainder selected by one reviewer.		☐ Yes<br>☐ No
6. Did the review authors perform data extraction in duplicate?		
For Yes, either ONE of the following:<br>☐ at least two reviewers achieved consensus on which data to extract from included studies<br>☐ OR two reviewers extracted data from a sample of eligible studies and achieved good agreement (at least 80 percent), with the remainder extracted by one reviewer.		☐ Yes<br>☐ No
7. Did the review authors provide a list of excluded studies and justify the exclusions?		
For Partial Yes:<br>☐provided a list of all potentially relevant studies that were read in full-text form but excluded from the review	For Yes, must also have:<br>☐ Justified the exclusion from the review of each potentially relevant study	☐ Yes<br>☐ Partial Yes<br>☐ No
8. Did the review authors describe the included studies in adequate detail?		
For Partial Yes (ALL the following):<br>☐ described populations ☐ described interventions ☐ described comparators ☐ described outcomes<br>☐ described research designs	For Yes, should also have ALL the following:<br>☐described population in detail<br>☐ described intervention in detail (including doses where relevant)<br>☐ described comparator in detail (including doses where	☐ Yes<br>☐ Partial Yes<br>☐ No

	relevant)<br>☐ described study's setting timeframe for follow-up	
9. Did the review authors use a satisfactory technique for assessing the risk of bias (RoB) in individual studies that were included in the review?		
**RCTs :**<br>For Partial Yes, must have assessed RoB from<br>☐unconcealed allocation, and<br>☐ lack of blinding of patients and assessors when assessing outcomes (unnecessary for objective outcomes such as all-cause mortality)	For Yes, must also have assessed RoB from:<br>☐ allocation sequence that was not truly random, and<br>☐ selection of the reported result from among multiple measurements or analyses of a specified outcome	☐ Yes<br>☐ Partial Yes<br>☐ No<br>☐ Includes only NRSI
**NRSI :**<br>For Partial Yes, must have assessed RoB:<br>☐ from confounding, and<br>☐ from selection bias	For Yes, must also have assessed RoB:<br>☐ methods used to ascertain exposures and outcomes, and ☐ selection of the reported result from among multiple measurements or analyses of a specified outcome	☐ Yes<br>☐ Partial Yes<br>☐ No<br>☐ Includes only RCTs
10. Did the review authors report on the sources of funding for the studies included in the review?		
For Yes<br>☐ Must have reported on the sources of funding for individual studies included in the review. Note: Reporting that the reviewers looked for this information but it was not reported by study authors also qualifies		☐ Yes<br>☐ No
11. If meta-analysis was performed did the review authors use appropriate methods for statistical combination of results?		
**RCTs**<br>For Yes:<br>☐ The authors justified combining the data in a meta-analysis<br>☐AND they used an appropriate weighted technique to combine study results and adjusted for heterogeneity if present.<br>☐ AND investigated the causes of any heterogeneity		☐ Yes<br>☐ No<br>☐ No meta-analysis conducted
**NRSI**<br>For Yes:		☐ Yes<br>☐ No

☐ The authors justified combining the data in a meta-analysis ☐ AND they used an appropriate weighted technique to combine study results, adjusting for heterogeneity if present<br>☐ AND they statistically combined effect estimates from NRSI that were adjusted for confounding, rather than combining raw data, or justified combining raw data when adjusted effect estimates were not available<br>☐ AND they reported separate summary estimates for RCTs and NRSI separately when both were included in the review	☐ No meta-analysis conducted
12. If meta-analysis was performed, did the review authors assess the potential impact of RoB in individual studies on the results of the meta-analysis or other evidence synthesis?	
For Yes:<br>☐ included only low risk of bias RCTs<br>☐ OR, if the pooled estimate was based on RCTs and/or NRSI at variable RoB, the authors performed analyses to investigate possible impact of RoB on summary estimates of effect.	☐ Yes<br>☐ No<br>☐ No meta-analysis conducted
13. Did the review authors account for RoB in individual studies when interpreting/ discussing the results of the review?	
For Yes:<br>☐ included only low risk of bias RCTs<br>☐ OR, if RCTs with moderate or high RoB, or NRSI were included the review provided a discussion of the likely impact of RoB on the results	☐ Yes<br>☐ No
14. Did the review authors provide a satisfactory explanation for, and discussion of, any heterogeneity observed in the results of the review?	
For Yes:<br>☐ There was no significant heterogeneity in the results<br>☐ OR if heterogeneity was present the authors performed an investigation of sources of any heterogeneity in the results and discussed the impact of this on the results of the review	☐ Yes<br>☐ No
15. If they performed quantitative synthesis did the review authors carry out an adequate investigation of publication bias (small study bias) and discuss its likely impact on the results of the review?	
For Yes:<br>☐ performed graphical or statistical tests for publication bias and discussed the likelihood and magnitude of impact	☐ Yes<br>☐ No

of publication bias	☐ No meta-analysis conducted
16. Did the review authors report any potential sources of conflict of interest, including any funding they received for conducting the review?	
For Yes:<br>☐ The authors reported no competing interests OR The authors described their funding sources and how they managed potential conflicts of interest	☐ Yes<br>☐ No

PICO: Population/Intervention /Comparator group/Outcome

RCT: Randomized controlled trial

NRSI: Non-randomized studies of the effects of interventions

Shea BJ, Reeves BC, Wells G, Thuku M, Hamel C, Moran J, Moher D, Tugwell P, Welch V, Kristjansson E, Henry DA. AMSTAR 2: a critical appraisal tool for systematic reviews that include randomized or non-randomized studies of healthcare interventions, or both. BMJ. 2017 Sep 21;358:j4008.

Table 3: Methodological quality assessment by AMSTAR 2

SRs	Item 1	Item 2	Item 3	Item 4	Item 5	Item 6	Item 7	Item 8	Item 9	Item 10	Item 11	Item 12	Item 13	Item 14	Item 15	Item 16	In Total of Yes	Overall quality
1 Zhang Y 2017	PY	N	N	PY	Y	Y	N	PY	Y	N	PY	N	Y	Y	Y	Y	7(43.75%)	Critically low
2 Meng D 2016	PY	N	N	PY	N	N	N	PY	Y	N	PY	N	N	N	Y	Y	3(18.75%)	Critically low
3 Liao MX 2017	Y	N	N	PY	N	Y	N	PY	N	N	PY	N	N	N	N	Y	2(12.5)	Critically low
4 Zhu Y 2012	Y	N	N	PY	Y	Y	N	PY	Y	N	PY	N	N	N	Y	N	4(25%)	Critically low
5 Hu TJ	PY	N	N	PY	N	Y	N	PY	Y	N	PY	N	Y	Y	Y	N	5(31.25%)	Critically

2015																	low
6 Chen J 2015	Y	N	N	PY	Y	Y	N	PY	N	N	PY	N	N	N	Y	Y	4(25%)	Critically low
7 He J 2009	Y	N	N	PY	N	Y	N	PY	Y	N	PY	N	Y	N	Y	Y	6(37.5%)	Critically low
8 Yuan ML 2011	Y	N	N	PY	N	N	N	PY	N	N	PY	N	N	Y	Y	Y	4(25%)	Critically low
9 Tang Q 2019	Y	N	N	PY	Y	Y	N	PY	N	N	PY	N	N	N	Y	Y	5(31.25%)	Critically low
10 Tang XR 2019	Y	N	N	PY	Y	Y	N	PY	Y	N	PY	N	Y	N	Y	Y	7(43.75%)	Critically low
11 Shi L 2018	PY	N	N	PY	Y	Y	N	PY	Y	N	PY	N	N	N	Y	Y	5(31.25%)	Critically low
12 Xu MH 2017	Y	N	N	PY	N	N	N	PY	Y	N	PY	N	N	Y	N	N	3(18.75%)	Critically low
13 Li S 2011	Y	N	N	PY	Y	Y	N	PY	Y	N	PY	N	Y	N	Y	Y	7(43.75%)	Critically low
14 Wang C 2017	Y	N	N	PY	N	N	N	PY	Y	N	PY	N	N	N	Y	N	3(18.75%)	Critically low
15 Tian Y 2014	Y	N	N	PY	Y	N	N	PY	N	N	PY	N	N	N	Y	Y	4(25%)	Critically low
16 Yu C 2016	Y	N	N	PY	Y	N	N	PY	Y	N	PY	N	N	N	Y	Y	5(31.25%)	Critically low
17 Wang LP 2006	Y	N	N	PY	N	Y	N	PY	N	N	PY	N	N	N	N	N	2(12.5)	Critically low
18 Li JP	Y	N	N	PY	N	Y	N	PY	Y	N	PY	N	N	N	Y	N	4(25%)	Critically

2016																	low
19 Liu H 2016	Y	N	N	PY	Y	Y	N	PY	Y	N	PY	N	N	N	Y	N	5(31.25%)	Critically low
20 Huang WX 2016	Y	N	N	PY	Y	Y	N	PY	Y	N	PY	N	N	N	Y	N	5(31.25%)	Critically low
21 Chen YY 2018	Y	N	N	PY	N	Y	N	PY	Y	N	PY	N	N	N	Y	N	4(25%)	Critically low
22 Xiang YX 2015	Y	N	N	PY	Y	Y	N	PY	Y	N	PY	N	N	N	Y	N	5(31.25%)	Critically low
23 Li LX 2019	Y	N	N	PY	Y	Y	N	PY	PY	N	Y	Y	Y	Y	Y	Y	9(56.25%)	low
24 Ye QP 2017	Y	N	N	PY	Y	Y	N	PY	PY	N	Y	Y	Y	N	N	Y	7(43.75%)	low
25 Long YB 2012	Y	N	N	PY	Y	Y	N	PY	PY	N	Y	Y	Y	Y	N	Y	8(50%)	low
26 S.Y. Wong 2012	Y	N	N	PY	Y	Y	N	PY	PY	N	No meta	No meta	N	Y	no meta	N	4(25%)	low
27 Li LX 2018	Y	N	N	PY	Y	Y	N	PY	PY	N	Y	Y	Y	Y	Y	Y	9(56.25%)	low
28 Xie Y 2008	Y	Y	N	Y	Y	Y	Y	PY	Y	N	no meta	no meta	Y	Y	no meta	N	9(56.25%)	moderate
29 Yang A 2016	Y	Y	N	Y	Y	Y	Y	PY	Y	N	Y	Y	Y	Y	Y	N	12(75%)	moderate
30 Bath PM 2018	Y	Y	N	Y	Y	Y	Y	PY	Y	Y	Y	Y	Y	Y	Y	N	13(81.25%)	moderate

31 Geeganage C 2012	Y	Y	N	Y	Y	Y	Y	PY	Y	N	Y	Y	Y	Y	Y	Y	13(81.25%)	moderate
**In total of "Y"**	27 (87.1%)	4 (12.9%)	0 (0%)	4 (12.9%)	21 (67.7%)	25 (80.6%)	4 (12.9%)	0 (0%)	20 (64.5%)	1 (3.2%)	7 (22.6%)	7 (22.6%)	13 (41.9%)	12 (38.7%)	24 (77.4%)	17(54.8%)		

SRs: Systematic reviews; Y: Yes; PY: Partial Yes; N: No; No meta: not conduct meta-analysis

Table 4: PRISMA checklist-2009

Section/topic	#	Checklist item
**TITLE**		
Title	1	Identify the report as a systematic review, meta-analysis, or both.
**ABSTRACT**		
Structured summary	2	Provide a structured summary including, as applicable: background; objectives; data sources; study eligibility criteria, participants, and interventions; study appraisal and synthesis methods; results; limitations; conclusions and implications of key findings; systematic review registration number.
**INTRODUCTION**		
Rationale	3	Describe the rationale for the review in the context of what is already known.
Objectives	4	Provide an explicit statement of questions being addressed with reference to participants, interventions, comparisons, outcomes, and study design (PICOS).

**METHODS**		
Protocol and registration	5	Indicate if a review protocol exists, if and where it can be accessed (e.g., Web address), and, if available, provide registration information including registration number.
Eligibility criteria	6	Specify study characteristics (e.g., PICOS, length of follow-up) and report characteristics (e.g., years considered, language, publication status) used as criteria for eligibility, giving rationale.
Information sources	7	Describe all information sources (e.g., databases with dates of coverage, contact with study authors to identify additional studies) in the search and date last searched.
Search	8	Present full electronic search strategy for at least one database, including any limits used, such that it could be repeated.
Study selection	9	State the process for selecting studies (i.e., screening, eligibility, included in systematic review, and, if applicable, included in the meta-analysis).
Data collection process	10	Describe method of data extraction from reports (e.g., piloted forms, independently, in duplicate) and any processes for obtaining and confirming data from investigators.
Data items	11	List and define all variables for which data were sought (e.g., PICOS, funding sources) and any assumptions and simplifications made.
Risk of bias in individual studies	12	Describe methods used for assessing risk of bias of individual studies (including specification of whether this was done at the study or outcome level), and how this information is to be used in any data synthesis.
Summary measures	13	State the principal summary measures (e.g., risk ratio, difference in means).
Synthesis of results	14	Describe the methods of handling data and combining results of studies, if done, including measures of consistency (e.g., I^2) for each meta-analysis.

**Section/topic**	**#**	**Checklist item**

Risk of bias across studies	15	Specify any assessment of risk of bias that may affect the cumulative evidence (e.g., publication bias, selective reporting within studies).
Additional analyses	16	Describe methods of additional analyses (e.g., sensitivity or subgroup analyses, meta-regression), if done, indicating which were pre-specified.
**RESULTS**		
Study selection	17	Give numbers of studies screened, assessed for eligibility, and included in the review, with reasons for exclusions at each stage, ideally with a flow diagram.
Study characteristics	18	For each study, present characteristics for which data were extracted (e.g., study size, PICOS, follow-up period) and provide the citations.
Risk of bias within studies	19	Present data on risk of bias of each study and, if available, any outcome level assessment (see item 12).
Results of individual studies	20	For all outcomes considered (benefits or harms), present, for each study: (a) simple summary data for each intervention group (b) effect estimates and confidence intervals, ideally with a forest plot.
Synthesis of results	21	Present results of each meta-analysis done, including confidence intervals and measures of consistency.
Risk of bias across studies	22	Present results of any assessment of risk of bias across studies (see Item 15).
Additional analysis	23	Give results of additional analyses, if done (e.g., sensitivity or subgroup analyses, meta-regression [see Item 16]).
**DISCUSSION**		
Summary of evidence	24	Summarize the main findings including the strength of evidence for each main outcome; consider their relevance to key groups (e.g., healthcare providers, users, and policy makers).
Limitations	25	Discuss limitations at study and outcome level (e.g., risk of bias), and at review-level (e.g., incomplete retrieval of identified research, reporting bias).
Conclusions	26	Provide a general interpretation of the results in the context of other evidence, and implications for future research.
**FUNDING**		

Funding	27	Describe sources of funding for the systematic review and other support (e.g., supply of data); role of funders for the systematic review.

From: Moher D, Liberati A, Tetzlaff J, Altman DG, The PRISMA Group (2009). Preferred Reporting Items for Systematic Reviews and Meta-Analyses: The PRISMA Statement. PLoS Med 6(7): e1000097. doi:10.1371/journal.pmed1000097. For more information, visit: **www.prisma-statement.org**.

Table 5: Reporting quality assessment by PRISMA

	SRs									
Item	1 Zhang Y 2017	2 Meng D 2016	3 Liao MX 2017	4 Zhu Y 2012	5 Hu TJ 2015	6 Chen J 2015	7 He J 2009	8 Yuan ML 2011	9 Tang Q 2019	10 Tang XR 2019
1	Y	Y	Y	Y	Y	Y	Y	Y	Y	Y
2	Y	Y	Y	Y	Y	Y	Y	Y	Y	Y
3	Y	Y	Y	Y	Y	Y	Y	Y	Y	Y
4	Y	Y	Y	Y	Y	Y	Y	Y	Y	Y
5	N	N	N	N	N	N	N	N	N	N
6	Y	Y	Y	Y	Y	Y	Y	Y	Y	Y
7	Y	Y	Y	Y	Y	Y	Y	Y	Y	Y
8	Y	N	Y	N	N	Y	Y	N	Y	N
9	Y	N	N	N	Y	Y	N	N	Y	Y
10	Y	N	Y	Y	Y	Y	Y	N	Y	Y
11	N	N	Y	N	Y	Y	N	N	Y	N
12	Y	N	Y	Y	N	N	Y	N	Y	Y
13	Y	Y	Y	Y	Y	Y	N	Y	Y	Y
14	Y	Y	Y	Y	Y	Y	Y	Y	Y	Y

15	Y	N	N	Y	Y	Y	Y	Y	Y	Y
16	Y	Y	N	Y	Y	Y	N	N	Y	Y
17	Y	Y	Y	N	Y	Y	Y	Y	Y	Y
18	Y	Y	Y	Y	Y	Y	Y	Y	Y	Y
19	Y	N	Y	Y	N	N	N	N	Y	Y
20	Y	Y	Y	Y	Y	Y	Y	Y	Y	Y
21	Y	Y	Y	Y	Y	Y	Y	Y	Y	Y
22	Y	N	N	Y	Y	Y	Y	Y	Y	Y
23	N	N	Y	Y	Y	Y	Y	N	N	Y
24	Y	Y	Y	Y	Y	Y	Y	Y	Y	Y
25	Y	Y	Y	N	N	N	N	Y	Y	N
26	Y	Y	Y	Y	Y	Y	Y	Y	Y	Y
27	Y	Y	Y	N	N	Y	Y	Y	Y	Y
In total of"Yes"	24(88.9%)	18(66.7%)	25(92.6%)	20(74.1%)	22(81.5%)	25(92.6%)	20(74.1%)	18(66.7%)	25(92.6%)	23(85.2%)

	SRs									
Item	11 Shi L 2018	12 Xu MH 2017	13 Li S 2011	14 Wang C 2017	15 Tian Y 2014	16 Yu C 2016	17 Wang LP 2006	18 Li JP 2016	19 Liu H 2016	20 Huang WX 2016
1	N	Y	Y	Y	Y	Y	Y	Y	N	N
2	Y	Y	Y	Y	Y	Y	Y	Y	Y	Y
3	Y	Y	Y	Y	Y	Y	Y	Y	Y	Y
4	Y	Y	Y	Y	Y	Y	Y	Y	Y	Y
5	N	N	N	N	N	N	N	N	N	N
6	Y	Y	Y	Y	Y	Y	Y	Y	Y	Y
7	Y	Y	Y	Y	Y	Y	Y	Y	Y	Y
8	N	N	N	Y	Y	N	Y	Y	Y	Y

9	Y	N	N	Y	Y	Y	N	Y	Y	Y
10	Y	Y	Y	Y	Y	N	N	Y	Y	Y
11	Y	N	Y	Y	N	N	N	Y	Y	Y
12	Y	N	N	N	Y	N	Y	Y	Y	Y
13	Y	Y	Y	Y	Y	Y	Y	Y	Y	Y
14	Y	Y	N	Y	Y	Y	Y	Y	Y	Y
15	N	N	Y	N	Y	Y	Y	Y	Y	N
16	N	N	Y	N	Y	N	Y	Y	Y	Y
17	Y	Y	Y	Y	Y	Y	N	Y	Y	Y
18	Y	N	Y	Y	Y	Y	Y	Y	Y	Y
19	N	N	N	N	Y	N	Y	Y	Y	Y
20	Y	Y	Y	Y	Y	Y	Y	Y	Y	Y
21	Y	Y	Y	Y	Y	Y	Y	Y	Y	Y
22	N	N	Y	N	Y	Y	Y	Y	Y	N
23	N	N	Y	Y	N	N	Y	Y	Y	Y
24	Y	Y	Y	Y	Y	Y	Y	Y	Y	Y
25	N	N	N	N	Y	Y	N	Y	Y	Y
26	Y	Y	Y	Y	Y	Y	Y	Y	Y	Y
27	Y	N	Y	N	Y	Y	N	N	N	N
In total of"Yes"	19(70.4%)	11(40.7%)	21(77.8%)	21(77.8%)	24(88.9%)	20(74.1%)	20(74.1%)	25(92.6%)	25(92.6%)	23(85.2%)

	SRs										
Item	21 Chen YY 2018	22 Xiang YX 2015	23 Li LX 2019	24 Ye QP 2017	25 Lon YB 2012	26 S.Y. Wong 2012	27 Li LX 2018	28 Xie Y 2008	29 Yang A 2016	30 Bath PM 2018	31 Geeganage C 2012
1	Y	Y	Y	Y	Y	Y	Y	Y	Y	Y	Y

2	Y	Y	Y	Y	Y	Y	Y	Y	Y	Y	Y
3	Y	Y	Y	Y	Y	Y	Y	Y	Y	Y	Y
4	Y	Y	N	N	N	N	N	N	Y	Y	Y
5	N	N	N	Y	N	N	Y	Y	Y	Y	Y
6	Y	Y	Y	Y	Y	Y	Y	Y	Y	Y	Y
7	Y	Y	Y	Y	Y	Y	Y	Y	Y	Y	Y
8	N	Y	Y	N	N	N	Y	Y	Y	Y	Y
9	Y	Y	Y	Y	Y	Y	Y	Y	Y	Y	Y
10	Y	Y	Y	Y	Y	Y	Y	Y	Y	Y	Y
11	Y	N	Y	N	Y	Y	Y	Y	Y	Y	Y
12	Y	Y	Y	Y	Y	Y	Y	Y	Y	Y	Y
13	Y	Y	Y	N	Y	N	Y	Y	Y	Y	Y
14	Y	Y	Y	N	Y	N	Y	Y	Y	Y	Y
15	N	Y	Y	N	Y	N	Y	Y	Y	Y	Y
16	Y	Y	Y	Y	Y	N	Y	Y	Y	Y	Y
17	Y	Y	Y	Y	Y	Y	Y	Y	Y	Y	Y
18	Y	Y	Y	Y	Y	Y	Y	Y	Y	Y	Y
19	Y	Y	Y	Y	Y	Y	Y	Y	Y	Y	Y
20	Y	Y	Y	Y	Y	N	Y	Y	Y	Y	Y
21	Y	Y	Y	Y	Y	N	N	Y	Y	Y	Y
22	N	Y	Y	N	Y	N	Y	Y	Y	Y	Y
23	Y	Y	Y	Y	Y	N	N	Y	Y	Y	Y
24	Y	Y	Y	Y	Y	Y	Y	Y	Y	Y	Y
25	Y	Y	Y	Y	Y	N	Y	Y	Y	Y	Y
26	Y	Y	Y	Y	Y	Y	Y	Y	Y	Y	Y
27	N	N	Y	Y	N	N	N	N	N	N	N

**In total of"Yes"**	**23(85.2%)**	**24(88.9%)**	**25(92.6%)**	**21(77.8%)**	**14(51.9%)**	**14(51.9%)**	**23(85.2%)**	**25(92.6%)**	**26(96.3%)**	**26(96.3%)**	**26(96.3%)**

	**Item of PRISMA**								
	**1**	**2**	**3**	**4**	**5**	**6**	**7**	**8**	**9**
**In total of "Yes"(31 SRs)**	28 (90.3%)	31 (100%)	31 (100%)	25 (80.6%)	6 (19.4%)	31 (100%)	31 (100%)	18 (58%)	23 (74.2%)
	**10**	**11**	**12**	**13**	**14**	**15**	**16**	**17**	**18**
**In total of "Yes"(31 SRs)**	27 (87.1%)	19 (61.3%)	23 (74.2%)	28 (90.3%)	28 (90.3%)	22 (71%)	23 (74.2%)	29 (93.5%)	30 (97%)
	**19**	**20**	**21**	**22**	**23**	**24**	**25**	**26**	**27**
**In total of "Yes"(31 SRs)**	21 (67.7%)	30 (97%)	29 (93.5%)	22 (71%)	21 (67.7%)	31 (100%)	20 (64.5%)	31 (100%)	14 (45.2%)

Y:Yes;N:No.
